# Complete Chloroplast Genome Sequencing and Phylogenetic Analysis of Two *Dracocephalum* Plants

**DOI:** 10.1155/2020/4374801

**Published:** 2020-12-29

**Authors:** Junjun Yao, Fangyu Zhao, Yuanjiang Xu, Kaihui Zhao, Hong Quan, Yanjie Su, Peiyu Hao, Jiang Liu, Benxia Yu, Min Yao, Xiaojing Ma, Zhihua Liao, Xiaozhong Lan

**Affiliations:** ^1^TAAHC-SWU Medicinal Plant Joint R&D Center, Tibetan Collaborative Innovation Center of Agricultural and Animal Husbandry Resources, Food Science College, Tibet Agriculture & Animal Husbandry University, Nyingchi, Tibet 860000, China; ^2^Key Laboratory of Forest Ecology in Tibet Plateau (Tibet Agricultural & Animal Husbandry University), Ministry of Education, Nyingchi, Tibet 860000, China; ^3^Chongqing Academy of Chinese Materia Medica, Chongqing 400065, China; ^4^State Key Laboratory of Dao-di Herbs, National Resource Center for Chinese Materia Medica, China Academy of Chinese Medical Sciences, Beijing 100700, China; ^5^Jiangxi Institute for Drug Control, NMPA Key Laboratory of Quality Evaluation of Traditional Chinese Patent Medicine, Nanchang, Jiangxi 330029, China; ^6^Key Laboratory of Eco-Environments in the Three Gorges Reservoir Region, Ministry of Education, Chongqing Engineering and Technology Research Center for Sweetpotato, School of Life Sciences, Southwest University, Chongqing 400715, China

## Abstract

*Dracocephalum tanguticum* and *Dracocephalum moldavica* are important herbs from *Lamiaceae* and have great medicinal value. We used the Illumina sequencing technology to sequence the complete chloroplast genome of *D. tanguticum* and *D. moldavica* and then conducted de novo assembly. The two chloroplast genomes have a typical quadripartite structure, with the gene's lengths of 82,221 bp and 81,450 bp, large single-copy region's (LSC) lengths of 82,221 bp and 81,450 bp, and small single-copy region's (SSC) lengths of 17,363 bp and 17,066 bp, inverted repeat region's (IR) lengths of 51,370 bp and 51,352 bp, respectively. The GC content of the two chloroplast genomes was 37.80% and 37.83%, respectively. The chloroplast genomes of the two plants encode 133 and 132 genes, respectively, among which there are 88 and 87 protein-coding genes, respectively, as well as 37 tRNA genes and 8 rRNA genes. Among them, the *rps2* gene is unique to *D. tanguticum*, which is not found in *D. moldavica*. Through SSR analysis, we also found 6 mutation hotspot regions, which can be used as molecular markers for taxonomic studies. Phylogenetic analysis showed that *Dracocephalum* was more closely related to *Mentha*.

## 1. Introduction

*Lamiaceae* is the sixth largest family of phanerogam. There are more than 7,000 species in about 220 genera and 10 subfamilies in the world. In China, there are over 800 species in 99 genera. This family is mainly distributed in Asia, Africa, and Europe. There are about 10 genera with more than 100 species, including *Salvia*, *Mesosphaerum*, and *Scutellaria*, among which the number of species accounts for about two-thirds of the species in this family. The plants of this family draw people's attention because of their aroma and medicinal value [1, 2], and they have great economic value. Among them, there are more than 60 kinds of plants in the *Dracocephalum*, which are mainly distributed in northern Asia, and a few extend to northern Europe or central Europe.

Chloroplast is a kind of plastid and one of the most important organelles in plants and algae cells. In addition to photosynthesis, chloroplasts also participate in the synthesis of starch, pigments, amino acids, and fatty acids [3–5]. Chloroplasts are green and oblate under a high-power microscope [6]. The currently accepted view on the origin of chloroplasts is the endosymbiosis theory. The chloroplasts in eukaryotic cells originated from endosymbiont cyanobacteria, which were engulfed and gradually digested by eukaryotic cells before becoming organelles in host cells through a long period of evolution [7]. As semiautonomous genetic organelles, chloroplasts have their own genetic material—chloroplast genome (cpDNA) [8, 9]. The length of the genome is about 120-160 kb, and the difference in the size of the chloroplast genome is mainly caused by the shrinkage and expansion of the IR region [10]. For example, the IR size of the chloroplast genome of *Pelargonium xhortorum* is 75,741 bp; the total length of its chloroplast genome is 217,942 bp [11]; the IR of *Pinus thunbergii* is only 495 bp, and its chloroplast genome is only 119,707 bp [12]; the chloroplast genome of *Metasequoia glyptostroboides* is much shorter due to loss of an IR region [13].

Genome sequencing is often used in the analysis of phylogenetic relationship and research on genetic diversity and evolution [14, 15]. The three independent genomes that can provide genetic information are chloroplasts, mitochondria, and cell nucleus. Compared with the complexity of the nuclear genome, the nonconservative nature of the large mitochondrial genome, the chloroplast genome is proper in size and easy for sequencing and acquisition [16–18]. Since 1986, the entire chloroplast genome of tobacco was first sequenced successfully [19]. To date, the NCBI GenBank database has collected the complete sequences of chloroplast genomes of more than 1,000 plants. The *Lamiaceae* family comprises about 200 species. In 2003, Paul Hebert introduced the concept of DNA barcoding, and gene sequencing technology began to be applied to biological species identification systems. Common DNA barcodes used by plants include *rbcL*, *matK*, *psbK-psbI*, *trnH-psbA*, and *ITS* [20]. However, using only a single gene for species identification may lead to different results from different genes, which brings about difficulties for species identification [21, 22]. Chloroplast genomes offer more variation than a single gene and will be more effective in addressing the evolutionary rules of close species.

In this study, we used Illumina sequencing technology to conduct full genome sequencing to the chloroplast genome of *D. tanguticum* and *D. moldavica* and then conducted de novo assembly and analysis. Moreover, based on the chloroplast genome data of *D. tanguticum* and *D. moldavica* and the chloroplast genomes of 30 other species, a phylogenetic tree was constructed. These data will help us better understand the evolutionary history of the branch of *Lamiaceae* and promote the research on the phylogeny, population, and genetic engineering of medicinal plants.

## 2. Results and Discussion

### 2.1. Characterization of Chloroplast Genomes in *Labiatae* Species

**Figure 1:**
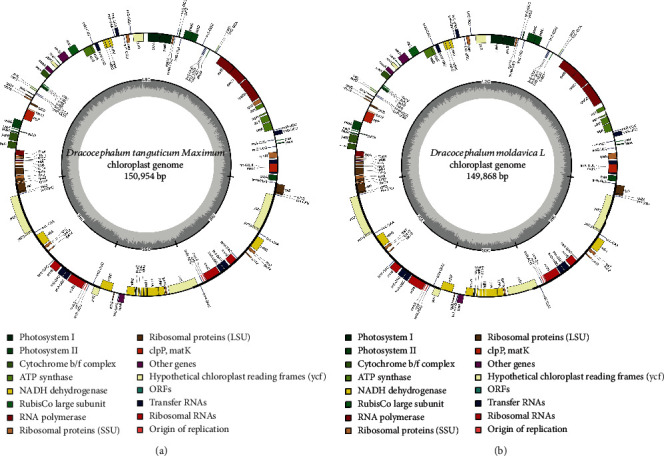
(a) *D. tanguticum* chloroplast genome. (b) *D. moldavica* chloroplast genome. The genes that code forward are on the outside of the circle, and the genes that code backward are on the inside. The legend identifies genes with different functions.

A total of 12.27 Gb raw data were obtained after sequencing. The chloroplast genome size, reads, and GC content of the two *Dracocephalum* plants are shown in Table 1. The chloroplast genome size of *Lamiaceae* plants is basically similar, which is about 150 kb. Through genome sequencing and de novo assembly, we obtained the complete chloroplast genome map of *D. tanguticum* and *D. moldavica* (Figure 1).

The total length of the chloroplast genomes of *D. tanguticum* and *D. moldavica* was 150,954 bp and 149,868 bp, respectively. Both *D. tanguticum* and *D. moldavica* contain a long single-copy region (LSC) with the lengths of 82,221 bp and 81,450 bp and a short single-copy region (SSC) with the lengths of 17,363 bp and 17,066 bp, respectively, which are separated by a pair of inverted repeat regions (IR) with the lengths of 51,370 bp and 51,352 bp, respectively (Table 1, Figure 1). The GC content of the chloroplast genome of these *Lamiaceae* plants is about 38%, and the GC content of the IR region, LSC region, and SSC region is about 43%, 36%, and 32%, respectively. *D. tanguticum* and *D. moldavica* encode 133 (114 unique genes) and 132 (113 unique genes) genes, respectively, including 37 tRNA genes and 8 rRNA genes, as well as 88 and 87 protein-coding genes, respectively. In addition, the *rps2* is a gene unique to *D. tanguticum*, which is not found in *D. moldavica* (Table 2). Gene deletions in other *Lamiaceae* species have also been reported before, and the deletion of the *trnK-UUU* gene in the chloroplast genome of *Pogostemon cablin* has ever been reported [23]. Generally speaking, the IR region is the most conservative region in the chloroplast genome [24]. The expansion and shrinkage of the IR, LSC, and SSC regions during evolution are the main reason for the difference in the length of the chloroplast genome [15, 25].

In addition, 18 genes were found to contain intron sequences, of which 16 genes contain one intron and 2 genes contain two introns. Among them, the intron in *trnK-UUU* is the largest and contains the *matK* gene (Table 3). The size of introns and gene spacers is also a factor that affects the size of the chloroplast genome, and intron loss has been reported in *Hordeum vulgare* [26, 27]. The two pseudogenes, *ycf1* and *rps19*, are located between IR and SSC and between IRa and LSC, respectively, which is consistent with the research in *Prunella vulgaris* [28]. CodonW software was used to calculate and analyze the codon usage bias (RSCU) of the chloroplast genome. The protein-coding sequences of the chloroplast genomes of *D. tanguticum* and *D. moldavica* consist of 26,754 and 26,460 codons, respectively. Among them, leucine (Leu) is used the most, and cysteine (Cys) is used the least. Such bias is considered to be a comprehensive result of natural selection, species mutation, and genetic drift [29, 30]. The complete chloroplast genome with gene annotations has been uploaded to the NCBI database, the accession numbers of *D. tanguticum* and *D. moldavica* are MT457746 and MT457747, respectively.

### 2.2. Comparative Analysis of Genome Structure

**Figure 2:**
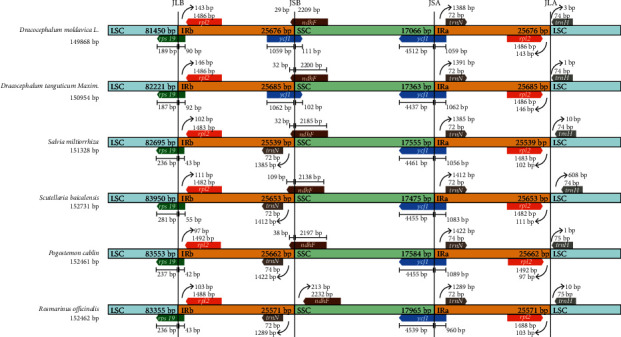
Comparison of the borders of LSC, SSC, and IR regions of chloroplast genomes in six *Lamiaceae* species.

The shrinkage and expansion of the boundary region between IR, LSC, and SSC are an important aspect of the chloroplast genome and are considered to be the main reason for the various sizes of the chloroplast genome [31, 32]. We analyzed the IR regions of *D. moldavica*, *D. tanguticum*, *S. miltiorrhiza*, *S. baicalensis*, *P. cablin*, and *R. officinalis*. The IR scopes of the six *Lamiaceae* plants are shown in Figure 2.

The *rps19* gene is located at the boundary of LSC/IRa. The size of the gene fragment in the LSC region is 187-281 bp, and the size of the gene fragment in the IRa region is 42-92 bp. The *rps19* and *ycf1* genes are usually cross-border genes, which is consistent with the reports on *Pongamia pinnata* [33]. The *rpl2* gene is located at the IR/LSC border, which is 1486 bp in both *D. tanguticum* and *D. moldavica*, but the distance from *rpl2* to the border in the *D. tanguticum* is 2 bp longer than that in the *D. moldavica*. Additionally, *ndhF* is also a gene that crosses the IRb/SSC border, but it was found in the analysis of the six *Lamiaceae* species that the *ndhF* gene in *Rosmarinus officinalis* is located in the SSC region, 213 bp away from the border (Figure 2), which is different from that of the other five *Lamiaceae* plants. Studies have shown that the small amplification of the IR region border is considered to be an important reason for maintaining the stability of the IR region [34]. Goulding et al. proposed the hypothesis of the evolution of the chloroplast's IR region: there is a boundary gene amplification mechanism in the IR region [35].

### 2.3. Analysis on Simple Repeat Sequences and Mutation Hotspots

We used vmatch v2.3.0 (http://www.vmatch.de/) software to identify the dispersed duplication sequences (Table 4). There were 36, 35 dispersed duplication sequences of 30-60 bp identified in *D. tanguticum* and *D. moldavica*, among which there are 18 forward repeats and 18, 17 palindrome repeats, respectively. No reverse and complementary repeat was found. Most of these repeats are between 30 and 40 bp. However, the dispersed duplication sequences of *D. tanguticum* and *D. moldavica* show great differences. For dispersed duplications between 40 and 60 bp, the forward and palindrome repetitions of *D. tanguticum* are evenly distributed, while the palindrome repetitions of *D. moldavica* are significantly more than the forward repetitions.

**Figure 3:**
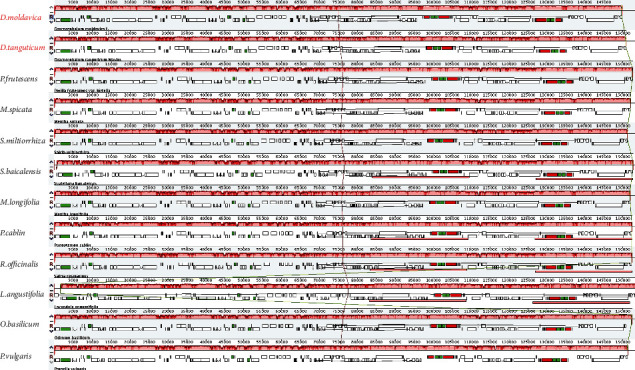
Chloroplast gene sequence alignment of 12 species of *Lamiaceae*. The rectangles represent the locations of genes in each genome. White represents CDs, green represents tRNA, and red represents rRNA.

The mauve software was used to compare the complete chloroplast genome of the two sequenced species with ten other *Lamiaceae* plants. Comparing the LCBs (local-collinear blocks) of these species, it was found that most of the genes maintained a consistent position and orientation, and the complete genome of the plastid showed a high degree of consistency, with no gene reversal detected. Through comparison of the complete chloroplast genome of 12 *Lamiaceae* plants, it was found that the *rpl2* gene was in the first location in the LSC region in *Lavandula angustifolia*, while the other 11 plants were in the last location in the IR region (Figure 3). This is different from predecessors' studies [36, 37]. The two differences can be used as the basis for species identification and molecular markers.

With MISA v1.0 (http://pgrc.ipk-gatersleben.de/misa/misa.html) software, we found 51 and 54 SSRs with at least 10 bp in *D. tanguticum* and *D. moldavica*, respectively (Table 5). Single-, double-, three-, and four-nucleotide SSRs were detected in *D. tanguticum* and *D. moldavica*, and one five-nucleotide SSR was also detected in *D. moldavica*. However, among them, single nucleotides in *D. tanguticum* and *D. moldavica* were 32 (62.75%) and 35 (64.81%), respectively, with a large proportion. Among SSRs of *D. tanguticum* and *D. moldavica*, the four-nucleotide repeat sequences are much more than three-nucleotide repeat sequences, which is consistent with the study of *Salvia miltiorrhiza* [38, 39].

Genes such as *rbcL*, *matK*, and *atpB* are widely applied as molecular markers in general phylogenetic studies [40]. After analysis of the SSRs of *D. tanguticum* and *D. moldavica*, we also found six mutation hotspot regions: *trnK-UUU*-*psb1*, *trnR-UCU*-*atpI*, *rpoC2*-*trnC-GCA*, *psaA*-*trnL-UAA*, *accD*-*cemA*, and *clpP*. Five of them are located in the intergenic region (IGS), and one is located in the intron region. Through comparison of the two *Dracocephalum* plants, the order of mutation rate from high to low is the noncoding region, the intron region, the LSC region, the SSC region, and the IR region.

### 2.4. Evolutionary and Phylogenetic Analysis

**Figure 4:**
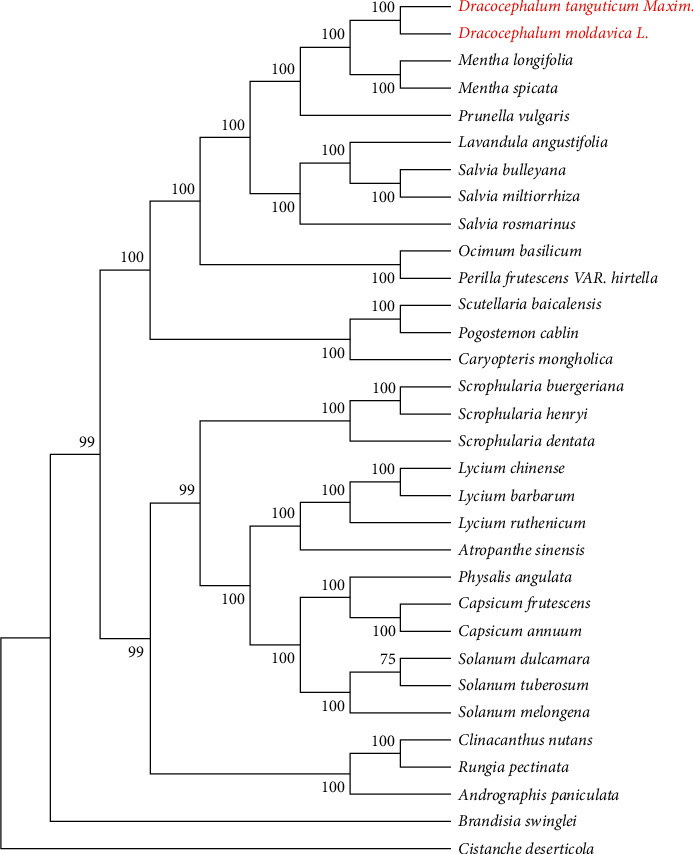
Phylogenetic tree constructed from the complete chloroplast genome.

In this study, we combined the sequenced chloroplast genomes of two *Dracocephalum* plants with that of 30 other angiosperms, constructed a phylogenetic tree with RAxML v8.2.10 (https://cme.h-its.org/exelixis/software.html) software based on the maximum likelihood (ML) method, and selected *Cistanche deserticola* (NC_021111.1) as the outgroup. Phylogenetic trees based on complete chloroplast genome data have a higher bootstrap value (the branch with a value greater than 75 is considered as a more stable branch). The results showed that *D. tanguticum* and *D. moldavica* belong to the same genus. Although the *D. tanguticum* is more similar to *Rosmarinus morphology*, the phylogenetic tree shows that the relationship between the two *Dracocephalum* plants and *Mentha* is closer than that with *Rosmarinus* (Figure 4). This result is consistent with traditional taxonomy. In the seed morphology, *Dracocephalum* is closer to *Mentha* [28, 41].

## 3. Materials and Methods

### 3.1. Material Acquisition

We used the leaves of *D. tanguticum* and *D. moldavica* for chloroplast genome sequencing. The vigorous, healthy, and fresh leaves of *D. tanguticum* and *D. moldavica* were obtained from the Science and Technology Park of Xizang Agriculture and Animal Husbandry College, Linzhi City, Tibet Autonomous Region, China. All leaves were immediately frozen in liquid nitrogen and stored at -80°C before analysis and use.

### 3.2. Sequencing and Assembly of Chloroplast Genomes

The modified CTAB (cetrimonium bromide) method was used to extract total genomic DNA from fresh leaves. Sheared low molecular weight DNA fragments were used to construct paired-end (PE) libraries according to the protocol of the Illumina manual (San Diego, CA, USA). Completed libraries were pooled and sequenced in the Illumina NovaSeq platform with PE150 sequencing strategy and 350 bp insert size (Genepioneer Biotechnologies Co. Ltd., Nanjing, Jiangsu, China). We used the clean data to assemble the chloroplast genomes with SPAdes v3.10.1 (http://cab.spbu.ru/software/spades/) software.

### 3.3. Annotation and Analysis of Chloroplast Genomes

CpGAVAS was used to annotate the sequence before manual correction. The blast v2.2.25 (https://blast.ncbi.nlm.nih.gov/Blast.cgi) software was used to compare the chloroplast genome's cds sequence on NCBI to correct cds; the hmmer v3.1b2 (http://www.hmmer.org/) software was used to correct the rRNA sequence; the aragorn v1.2.38 (http://130.235.244.92/ARAGORN/) software was used to correct the tRNA. The OGDRAW program (https://chlorobox.mpimp-golm.mpg.de/OGDraw. html) was used to make a circular map of the chloroplast genome. The base content was analyzed with biological editing software, and the nucleotide diversity (RSCU) was analyzed with MEGA 7 software.

We used REPuter (http://bibiserv.techfak.uni-bielefeld.de/ter/) to look for and analyze the size and position of forward, reverse, palindrome, and complementary repeat sequences. MISA (http://pgrc.ipk.gatersleben.de/misa/) was adopted to identify SSRs, the threshold for single nucleotide SSRs is 10 repeats, the threshold for double-nucleotide SSRs is 5 repeats, the threshold for three-nucleotide SSRs is 4 repeats, and the threshold of four-, five-, and six-nucleotide SSRs is 3 repeats. The mauve software was used to sequence alignment of the chloroplast genome.

### 3.4. Phylogenetic Analysis

Based on the chloroplast genome data of *D. tanguticum* and *D. moldavica* and the chloroplast genomes of 30 other species, a phylogenetic tree was constructed, and *Cistanche deserticola* (NC_021111.1) chloroplast genome was selected as the outgroup. Before constructing the phylogenetic tree, we used MAFFT software (V7.427, auto mode) for multisequence alignment to obtain neatly arranged chloroplast genomes for phylogenetic analysis. By default, the whole genome is used for evolutionary tree analysis, and the ring sequence is set at the same starting point. MAFFT software was used to perform multiple sequence alignment between species sequences, and trimAl (v1.4.rev15) was used to prune the aligned data. Then, RAxML V8.2.10 (https://cme.h-its.org/exelixis/software.html) software was used, its GTRGAMMA model was selected for Bootstrap analysis, and the Bootstrap = 1000 was set to build the maximum likelihood evolutionary tree.

## 4. Conclusion

The size, GC content, gene order, and number of chloroplast genomes in *D. tanguticum* and *D. moldavica* are highly similar. As *D. moldavica* lacks the *rps2* gene compared to *D. tanguticum*, which may be one of the reasons that *D. moldavica* is 1,086 bp shorter than *D. tanguticum* in the full length of the chloroplast genome. Through SSR analysis, six mutation hotspot regions were found, among which five were located in the intergenic region, and one was located in the intron region. Through phylogenetic tree analysis, it was found that *Mentha* and *Dracocephalum* have a closer genetic relationship. In this study, we have provided the complete chloroplast genome of *D. tanguticum* and *D. moldavica*, which provides a basis for the identification and overcoming of phylogenetic problems at the species level.

## Figures and Tables

**Table 1:** Chloroplast genome information of six species of *Lamiaceae*.

Genome features	*D. tanguticum*	*D. moldavica*	*S. miltiorrhiza*	*P. cablin*	*S. baicalensis*	*P. officinalis*
Genome size (bp)	150,954	149,868	151,328	152,460	151,824	152,462
LSC size (bp)	82,221	81,450	82,695	83,974	83,976	83,355
SSC size (bp)	17,363	17,066	17,555	17,652	17,338	17,969
IR size (bp)	51,370	51,352	51,078	50,834	50,510	51,138
Number of genes (unique)	133 (114)	132 (113)	131 (114)	126 (109)	132 (114)	132 (113)
Protein genes (unique)	88 (8)	87 (8)	86 (6)	90 (8)	87 (7)	94 (9)
tRNA genes (unique)	37 (7)	37 (7)	37 (7)	28 (5)	37 (7)	30 (6)
rRNA genes (unique)	8 (4)	8 (4)	8 (4)	8 (4)	8 (4)	8 (4)
Duplicated genes in IR	19	19	17	17	18	19
GC content (%)	37.80%	37.83%	38.00%	38.24%	38.30%	38.00%
GC content in LSC (%)	35.84%	35.81%	35.90%	36.30%	36.30%	36.20%
GC content in SSC (%)	31.85%	31.79%	32.00%	32.10%	32.70%	31.90%
GC content in IR (%)	43.04%	43.03%	43.10%	43.50%	43.60%	43.00%
Total reads	21517059	19384791	—	—	—	—
Aligned paired-end reads	1531708	2709906	—	—	—	—
Average organelle coverage	3033	5412	—	—	—	—
Average insert size (bp)	323 ± 60	331 ± 63	—	—	—	—

**Table 2:** Annotated gene list of chloroplast genomes of *D. tanguticum* and *D. moldavica*.

Category of gene	Group of gene	Name of gene
Photosynthetic	Subunits of photosystem I	*psaA*, *psaB*, *psaC*, *psaI*, *psaJ*
	Submits of photosystem II	*psbA*, *psbB*, *psbC*, *psbD*, *psbE*, *psbF*, *psbH*, *psbI*, *psbJ*, *psbK*, *psbL*, *psbM*, *psbN*, *psbT*, *psbZ*
	Subunits of NADH dehydrogenase	*ndhA*, *ndhB(x2)*, *ndhC*, *ndhD*, *ndhE*, *ndhF*, *ndhG*, *ndhH*, *ndhI*, *ndhJ*, *ndhK*
	Subunits of cytochrome b/f complex	*petA*, *petB*, *petD*, *petG*, *petL*, *petN*
	Subunits of ATP synthase	*atpA*, *atpB*, *atpE*, *atpF*, *atpH*, *atpI*
	Large subunit of rubisco	*rbcL*

Self-replication	Proteins of large ribosomal subunit	*rpl2(x2)*, *rpl14*, *rpl16*, *rpl20*, *rpl22*, *rpl23(x2)*, *rpl32*, *rpl33*, *rpl36*
	Proteins of small ribosomal subunit	*rps2* ^∗^, *rps3*, *rps4*, *rps7(x2)*, *rps8*, *rps11*, *rps12(x2)*, *rps14*, *rps15*, *rps16*, *rps18*, *rps19*
	Subunits of RNA polymerase	*rpoA*, *rpoB*, *rpoC1*, *rpoC2*
	Ribosomal RNAs	*rrn23S(x2)*, *rrn16S(x2)*, *rrn5S(x2)*, *rrn4.5S(x2)*
	Transfer RNAs	*trnA-UGC(x2)*, *trnH-GUG*, *trnK-UUU*, *trnQ-UUG*, *trnS-GCU*, *trnS-UGA*, *trnS-GGA*, *trnG-UCC*, *trnG-GCC*, *trnR-UCU*, *trnR-ACG(x2)*, *trnC-GCA*, *trnD-GUC*, *trnY-GUA*, *trnE-UUC*, *trnT-GGU*, *trnT-UGU*, *trnfM-CAU*, *trnL-UAA*, *trnL-CAA(x2)*, *trnL-UAG*, *trnF-GAA*, *trnM-CAU*, *trnV-UAC*, *trnV-GAC(x2)*, *trnW-CCA*, *trnP-UGG*, *trnI-CAU(x2)*, *trnI-GAU(x2)*, *trnN-GUU(x2)*

Biosynthesis	Maturase	*matK*
	Protease	*clpP*
	Envelope membrane protein	*cemA*
	Acetyl-CoA carboxylase	*accD*
	c-type cytochrome synthesis gene	*ccsA*
	Translation initiation factor	*infA*

Unknown function	Conserved hypothetical chloroplast Reading frames	*ycf1(x2)*, *ycf2(x2)*, *ycf3*, *ycf4*, *ycf15(x2)*

^∗^Represents the gene unique to *D. tanguticum*.

**Table 3:** Intron information of *D. tanguticum* and *D. moldavica*.

Gene	Location	Exon I (bp)	Intron I (bp)	Exon II (bp)	Intron II (bp)	Exon III (bp)
*trnK-UUU*	LSC	37	2544	35		
*rps16*	LSC	42	869	225		
*trnG-UCC*	LSC	23	682	47		
*atpF*	LSC	152	706	385		
*rpoC1*	LSC	456	766	1620		
*trnL-UAA*	LSC	38	498	49		
*trnV-UAC*	LSC	35	575	38		
*rps12*	LSC	114	536	258		
*petB*	LSC	7	724	641		
*petD*	LSC	8	708	475		
*rpl16*	LSC	9	874	399		
*rpl2*	IR	391	661	434		
*ndhB*	IR	777	677	756		
*trnI-GAU*	IR	37	947	35		
*trnA-UGC*	IR	38	801	35		
*ndhA*	SSC	553	1004	539		
*clpP*	LSC	71	691	292	646	228
*ycf3*	LSC	129	705	228	746	153

**Table 4:** *D. tanguticum* and *D. moldavica* dispersed duplication sequence information.

Total numbers	Forward	Palindromic	Total regions	IGS	Introns	Coding region
36	18	18	18	9	2	7
35	18	17	17	6	3	8

**Table 5:** *D. tanguticum* and *D. moldavica* SSR information.

Species	Total	P1	P2	P3	P4	Pc	LSC	SSC	IR
*D. tanguticum*	51	32	5	1	2	11	44	3	2 (2)
*D. moldavica*	54	35	6	1	3	9	45	5	2 (2)

P1: single-nucleotide; P2: double-nucleotide; P3: three-nucleotide; P4: four-nucleotide; Pc: complex-nucleotide.

## Data Availability

The complete chloroplast genome data used to support the findings of this study have been deposited in the NCBI repository (the accession numbers of *D. tanguticum* and *D. moldavica* are MT457746 and MT457747, respectively).
